# Case Report: Imaging features of a new type double inferior vena cava malformation and review

**DOI:** 10.3389/fcvm.2023.1298071

**Published:** 2023-12-04

**Authors:** Yu-lin Wu, Guo-kai Yang, Qian Chen, Yi Tang, Jian-hui Zhang, Shao-jie Wu, Sen-lin Cai, Yan-feng Zhou, Yao-Bin Zhu, Jie-wei Luo, Zhu-ting Fang

**Affiliations:** ^1^ Fujian Provincial Hospital, Shengli Clinical Medical College of Fujian Medical University, Fuzhou, China; ^2^ Department of Interventional Radiology, Fujian Provincial Hospital, Fuzhou, China; ^3^ Department of Traditional Chinese Medicine, The First Affiliated Hospital, Fujian Medical University, Fuzhou, China; ^4^ Department of Traditional Chinese Medicine, Fujian Provincial Hospital, Fuzhou, China

**Keywords:** double inferior vena cava, congenital abnormalities, computed tomography venography, inferior vena cava filter, venous thromboembolism

## Abstract

**Background:**

Double inferior vena cava (DIVC) is a rare vascular malformation. With advances in radiological techniques and diagnosis, more and more types of DIVC were identified and diagnosed. Recognition of the variety of DIVC seen on imaging is essential for subsequent venous interventions.

**Case presentation:**

A 77-year-old man presented with low back pain with left lower limb pain for 1 month. Scattered petechiae above the skin surface on the left lower leg, especially on the extensor surface, with flaking and mild tingling of the skin, were noted 3 weeks ago. Ultrasound revealed deep vein thrombosis (DVT) in the left lower limb. Computed tomography pulmonary angiography (CTPA) suggested no significant thrombus in the pulmonary artery. Computed tomography venography (CTV) of bilateral lower limbs showed that iliac vein compression syndrome with formation of deep and superficial venous traffic branches in bilateral lower limbs, predominantly on the left side. CTV of the inferior vena cava (IVC) suggested that the left common iliac vein crossed the common iliac artery bifurcation from dorsal to ventral and continued to travel cranially as a ventral vessel, and connected with the ventral IVC anterior to the right common iliac artery. The right common iliac vein extended cephalad as a dorsal vessel, which was narrowed at the level of 4th lumbar vertebra by compression of the hyperplastic bone and the osteophyte. The patient was discharged after right and left common iliac vein angiography and balloon dilation of bilateral common iliac vein.

**Conclusion:**

The formation of both ventrally and dorsally aligned DIVC is rarer. It should be clarified the effects of DIVC on DVT formation, and the importance of imaging for preoperative planning.

## Introduction

Inferior vena cava (IVC), the largest venous trunk in the body, is the main trunk of the IVC system. IVC is formed by the confluence of bilateral common iliac veins at the level of the 5th lumbar vertebra, rising along the right side of the abdominal aorta, entering the thoracic cavity through the vena cava orifice of the diaphragm and into the right atrium. It collects venous blood from the lower limbs, pelvis and abdomen. Congenital anomalies of IVC are rare (occurring in 0.3% of healthy individuals and 0.6% of individuals with other cardiovascular diseases), with double inferior vena cava (DIVC) being a relatively rare congenital abnormalities, accounting for 0.2%–3.0% of the general population (1, 2). In this article, we present a rare case of DIVC and review its various types.

## Case presentation

A 77-year-old man was admitted with a complaint of low back pain accompanied by left lower limb pain for 1 month. A month ago, the patient presented low back pain with no apparent cause, mainly in the left sciatic nerve distribution, accompanied by weakness in the left lower limb and numbness in the left calf and foot, which did not ease with time, change of position, or rest. His medical history included the loss of the end of his left index finger due to a car accident five years ago. Three weeks ago he had his blood pressure measured at the local hospital at 180/90 mm Hg without a history of hypertension and taking antihypertensive agents. Magnetic Resonance (MR) of the lumbar spine revealed herniated discs at 3rd to 4th and 4th to 5th lumbar vertebrae. After management with Mannitol to reduce neuroedema, Cytosine to improve circulation and Lofenestine tablets to relieve pain, symptoms were not relieved. Scattered petechiae above the skin surface on the left lower leg, especially on the extensor surface, with flaking and mild tingling of the skin, were noted three weeks ago.

**Figure 1:**
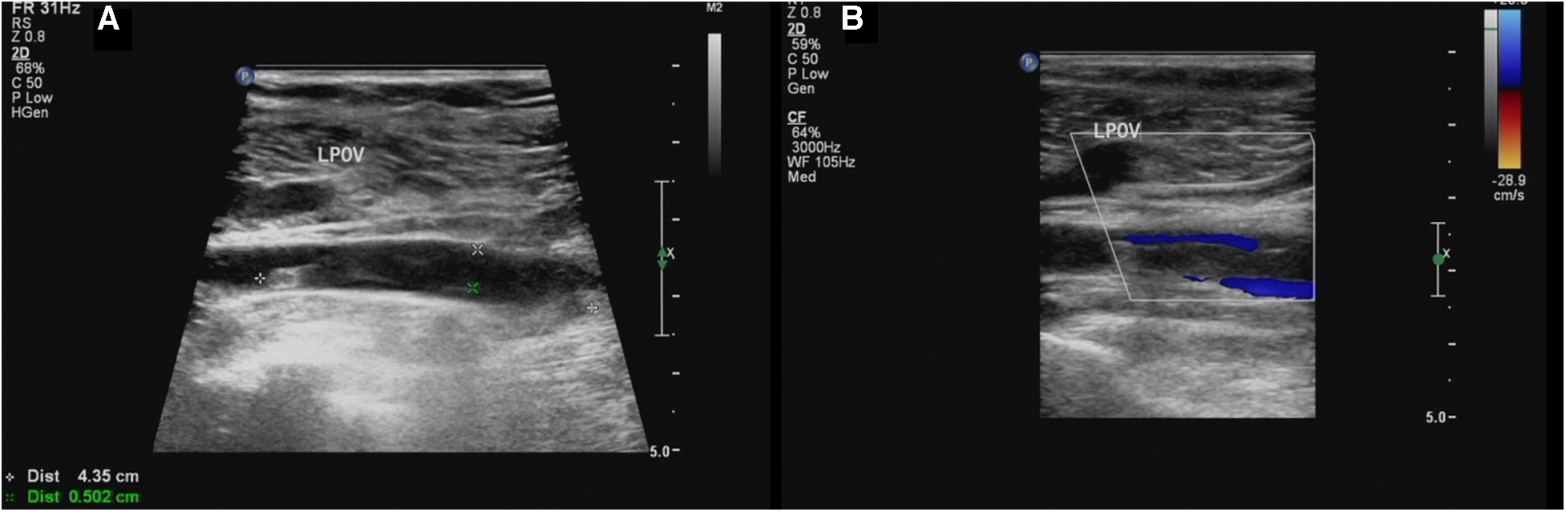
(**A,B**) Sagittal sonogram and sagittal color Doppler US limb of thrombosis of the left popliteal vein (LPOV).

Our ultrasound showed left lower extremity deep vein thrombosis (DVT), including a thrombus in the popliteal vein measuring approximately 43.5mm × 5.0 mm (Figure 1). Electromyography revealed no neurogenic or myogenic injury in either lower limb. Computed tomography pulmonary angiography (CTPA) showed no signs of thrombosis in the pulmonary artery and severe stenosis of the proximal lumen of the abdominal trunk.

**Figure 2:**
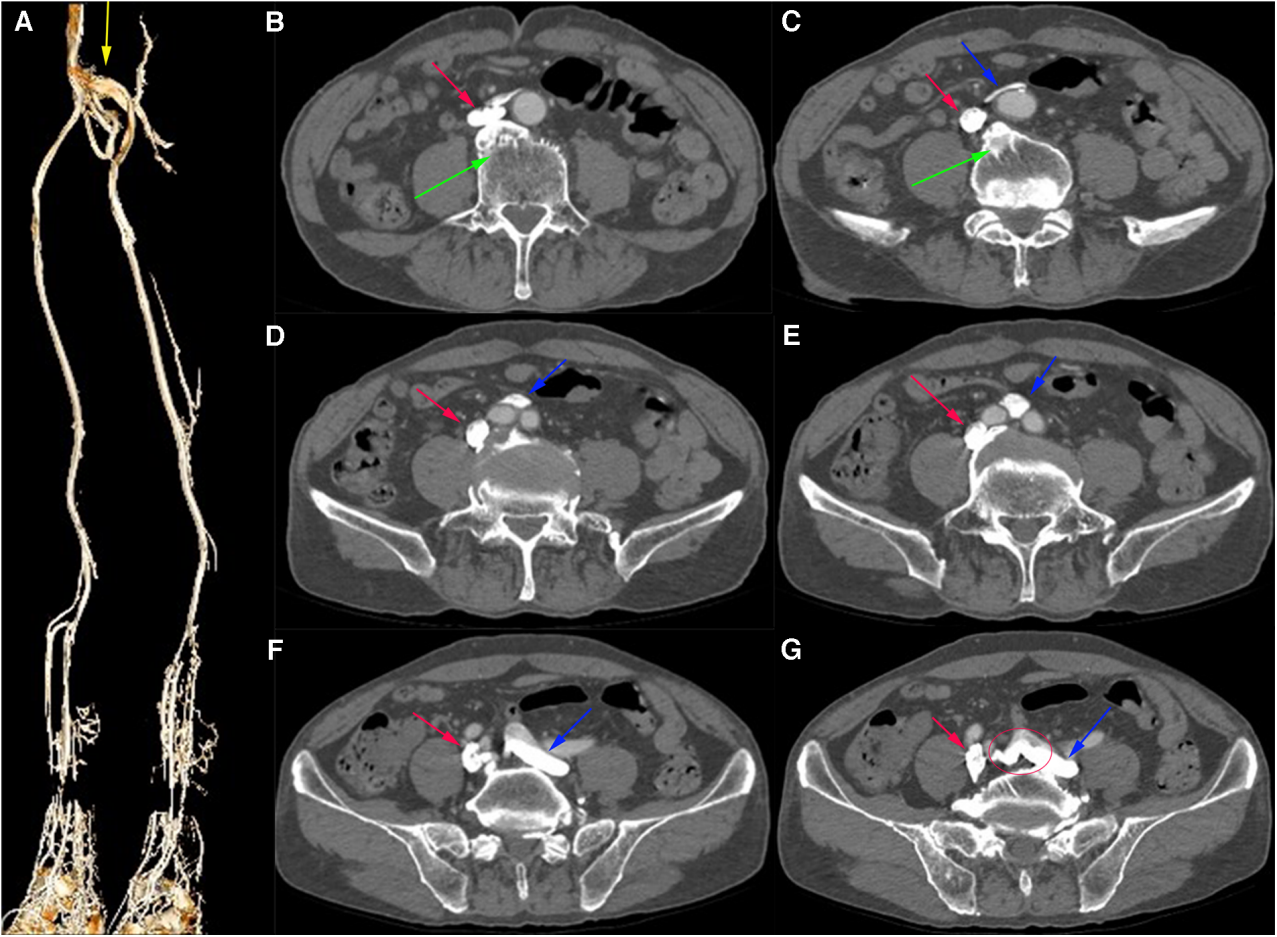
(**A**) Virtual Reality (VR) image of the patient's pre-intervention CTV of lower limb, with anastomosing branches seen between the iliac veins bilaterally. (**B**–**G**) Axial venous phase image, with osteophytes seen (green arrows) and the left common iliac vein (blue arrow) bypassing the abdominal aorta ventrally and traveling dorsally from between the right and left common iliac arteries, with anastomosing branches seen between the common iliac veins.

**Figure 3:**
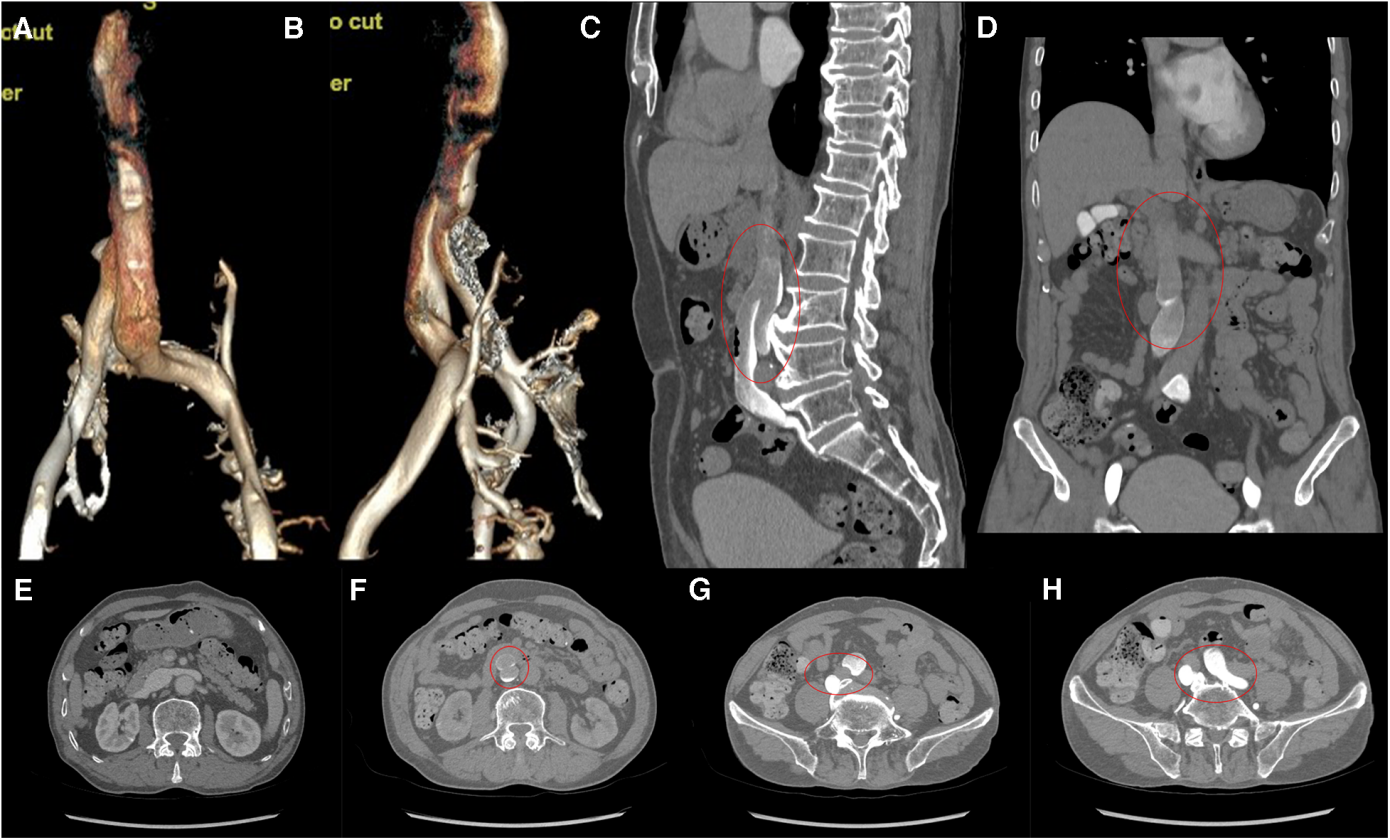
(**A,B**) VR images of CTV of IVC. (**C,D**) MPR images of the sagittal and coronal venous phases of IVC, showing DIVC in a ventral-dorsal relationship (red oval), confluent at the level of 3rd lumbar vertebra. (**E**) Plain scan, with normal development of the bilateral renal veins where they converge into IVC. (**F**–**H**) Images of the axial venous phase, with the inferior renal segment of the IVC divided into two ventral and dorsal branches. The left common iliac vein crosses between the bilateral common iliac arteries to join the ventral vessel, and the right common iliac vein joins the dorsal vessel.

Computed tomography venography (CTV) revealed bilateral lower limb iliac vein lumen stenosis with paravalvular formation around and in front of the sacrum (Figure 2). Distorted and thickened vascular shadows were seen in the bilateral calf muscle spaces, especially on the left side. Then, CTV of IVC suggested that the left common iliac vein traveled cranially and crossed the bifurcation of the common iliac artery, and continued cephalad as a ventral vessel, while the right common iliac vein extended cephalad as a dorsal vessel. The dorsal vessel was narrowed at the level of 4th lumbar vertebrae, by the compression of the hyperplastic bone and the osteophyte (Figure 3). Both vessels run parallel to the abdominal aorta and converged on the right side of the abdominal aorta, at a level where bilateral renal veins converge 12.5 mm pedal to the IVC. The suprarenal segment, renal segment and bilateral renal veins of the IVC are normal.

**Figure 4:**
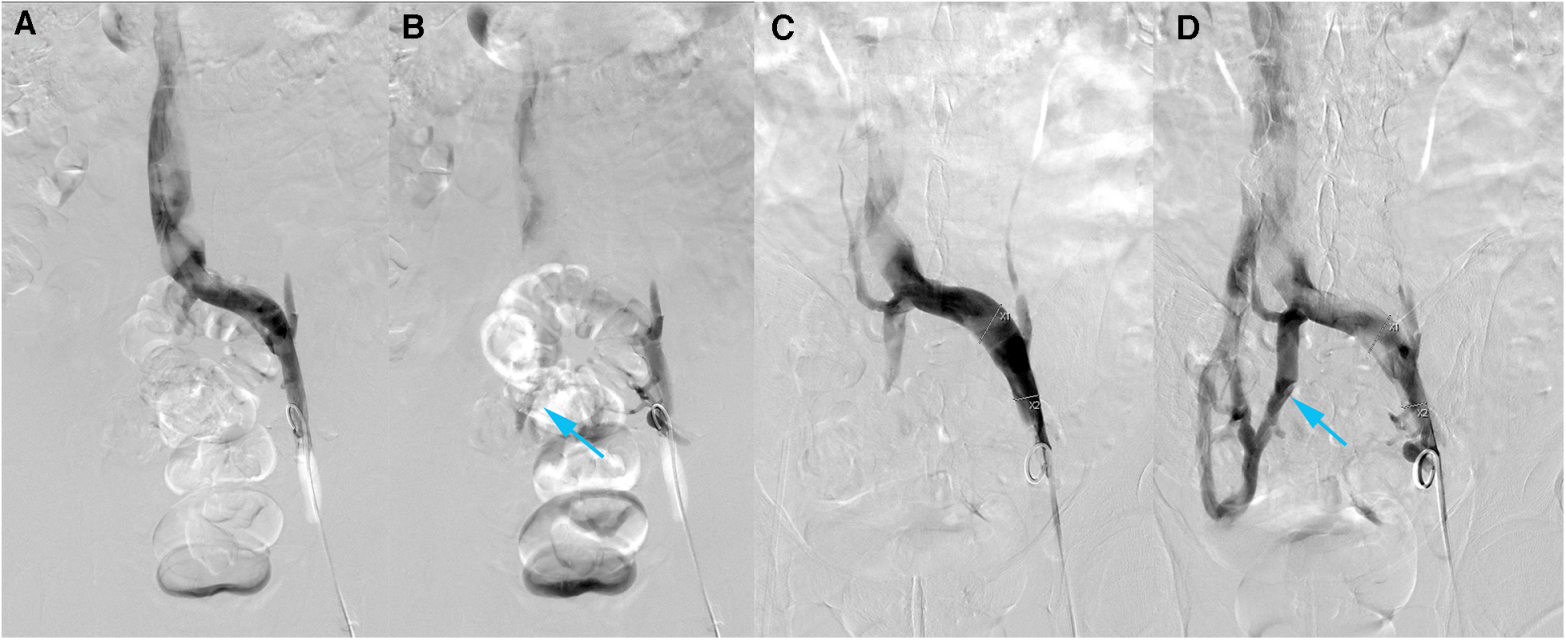
DSA of left iliac vein. (**A,B**) Before intervention. (**A**) Early phase AP. (**B**) Late phase AP. (**C,D**) After intervention. (**C**) Early phase AP. (**D**) Late phase AP. Visible collateral vessels formation (blue arrow). After balloon dilatation, the visualization of collateral vessels have been more obvious.

Bilateral femoral vein was punctured under local anesthesia and a 5F catheter was sent for bilateral iliac venography with Posterior Anterior (PA) and Left Anterior Oblique (LAT). A 12 mm × 8 cm balloon was used to dilate the right and left common iliac veins and repeated the left and right iliac venograms. A total of about 120 ml of iodixanol was used and the operation went smoothly without adverse effects. Digital Subtraction Angiography (DSA) suggested stenosis of the left common iliac vein, approximately 50%–80%. After balloon dilatation, the stenosis was improved, and the visualization of collateral vessels was significantly enhanced (Figure 4). The lumen of the right external iliac vein and right common iliac vein is still patent, with no significant stenosis or obstructive lesions. The distal segment of the IVC appears to have a limited defect. After discharge, the patient was treated with long-term anticoagulation.

## Discussion

DIVC is a rare congenital variant of IVC, and the earliest report of inferior vena cava duplication was in 1912 (3). Most of the subsequent studies were due to incidental findings during imaging examinations for other diseases. At 4–8 weeks of age, the inferior hepatic vena cava originates from a group of three pairs of parallel veins, which, in chronological order of formation, are the posterior cardinal, subcardinal, and supracardinal veins (4, 5). Variations in the development of IVC are attributed to misalignment of the anastomosis, the degeneration of structures that should persist, and conversely, the persistence of structures that should degenerate (6). Normal IVC is composed of four segments: hepatic, suprarenal, renal, and infrarenal, each of which has a different embryonic origin. The embryonic development of IVC is a complex process involving the development, regression, anastomosis, and replacement of the three main veins during the embryonic period. The posterior cardinal vein first appears in the posterior part of the embryo. Subsequently, the subcardinal vein appears anterior and medial to the posterior main vein. The right subcardinal vein is still present, forming the suprarenal segment of IVC, while the left subcardinal vein completely degenerates. Then, the supracardinal vein appears dorsal to the subcardinal vein and the right supracardinal vein forms the infrarenal segment of the IVC. The renal segment is the anastomosis between right subcardinal and right supracardinal, while the left supracardinal vein degenerates (7, 8). The vitelline vein originates from the capillary plexus of the yolk sac and forms the intrahepatic segment. The thoracic segment of the right supracardinal vein forms the odd vein and the caudal end forms the lumbar vein, while the caudal end of the posterior main vein persists as the common iliac vein. The caudal end of the subcardinal vein degenerates to form the gonadal vein. The basal interstitial vein forms the left renal vein (9). Bilateral iliac veins originate from the posterior cardinal vein, which anastomoses between them form a confluence of common iliac veins behind the pelvic aorta (10, 11).

We presume that DIVC can be divided into two broad categories: bilateral and ipsilateral. In previous studies, there were three types of IVC duplication: Type I- bilaterally symmetrical trunks and an approximately equal-sized the preaortic trunk; Type II-bilaterally symmetrical trunks of approximately the same size but smaller than the preaortic trunk; Type III-disproportionate left and right IVCs (left IVC smaller in diameter than right IVC) and unequal size compared to the preaortic trunk (6, 12). This is a bilateral DIVC classification. In terms of size, the present case appears to be a subtype of Type II. If bilateral supracardinal veins persist during development, two distinct IVC will form. The left IVC drains to the left renal vein and then across intermediate line to the right. If the upper renal IVC is dilated beyond the upper limit of the filter-compatible vessels, placing the filter in the lower renal IVC is preferable (5, 13, 14). Doyle et al. (10) first described an ipsilateral repeat of the IVC. Kim et al. (15) considered right DIVC to be a complex developmental anomaly and not a double inferior vena cava developmental variant, as it is often combined with other congenital variants, e.g., posterior vena cava ureter and anterior left iliac vein of the aorta. In our case, the right ureter runs normally. Several studies have reported DIVC with interiliac vein formation, which is consistent with our case (16, 17). DIVC can also be classified according to the segment in which it occurs. In this case, it occurs in the infrarenal segment and is all located on the right side of the aorta, which can be referred to as the right infrarenal DIVC. We presume that the ventral IVC originates from the right subcardinal vein, and the dorsal IVC originated from the right supracardinal vein, forming the right DIVC. The ventral branch of the periaqueductal venous ring forms the left common iliac vein and connects to the ventral IVC anterior to the right common iliac artery. The dorsal IVC passes posterior to the right common iliac artery and continues into the right common iliac vein. Previous studies have attributed this to the fact that the right subcardinal vein is closer to the ventral side and that the right gonadal vein, which originates from the right subcardinal vein, joins the ventral vessel (18, 19). In the case they reported, there was no confluence of iliac veins or traffic veins between bilateral iliac veins, which is inconsistent with our case. Meyer et al. (20) suggested that the ventral vessel originate from the right subcardinal vein because the diameter is smaller than that of the dorsal vessel. In our case, the diameters were similar, which is not suitable for discussing the origin of the vessels. In our report, this type of DIVC had three anatomical features: (1) Two IVC in a ventral-dorsal relationship; (2) Confluence or traffic veins between the bilateral iliac veins; (3) without ureter located posterior to the inferior vena cava.

The complex development of the IVC may result in anatomical anomalies which can impede venous return and stimulate thrombosis. Most previous studies have reported dysplasia as the most common type of IVC anomaly causing deep vein thrombosis (DVT). Sitwala et al. (21) identified congenital IVC deficiency as a risk factor for lower limb deep vein thrombosis in young people. Kim et al. (22) researched the types of abnormal development of the inferior vena cava in the normal population and in patients with DVT, and they reported that agenesis and hypoplasia of the inferior vena cava were the most common types in patients with DVT. In our patient, the dorsal vessels were constricted by lumbar hyperosteogeny, which led to impaired venous drainage and increased venous pressure distal to the stenosis. However, there is no obvious thrombosis in the vein of the right lower extremity, but thrombosis can be seen on the left. Hence, we suggest that this type of DIVC is not correlated with DVT.

The placement of an IVC filter is a intervention for the prevention of pulmonary artery embolism (PAE) in patients with DVT. IVC filters are usually placed below the level of the renal vein using transfemoral or transjugular veins, both of which can also be used to retrieve filters. Proper radiological diagnosis of DIVC is essential for filter placement. When PAE occurs after filter placement, the presence of DIVC should be suspected. Previous studies have reported interventions in patients with repeated DVT and/or pulmonary embolism in the bilateral inferior vena cava using either one filter in the left and one in the right IVC or coil embolization or transcatheter thrombolysis on the side with the larger vessel diameter (23–25). In addition to the above two treatments, a single filter can be placed after a repeat IVC confluence, depending on the distance from the confluence to the level of the renal vein confluence and its canal diameter. Lei Jiang et al. (26) reported that when the confluence is above the level of the renal vein, there is no need to place a filter on one side if no DVT is found on that side.

Therefore, imaging is particularly important for the correct diagnosis of IVC. CTV is the most commonly used test for initial identification of DIVC, usually using the portal phase (60–70s evaluation of IVC after intravenous administration of 120–150 ml of contrast medium at a rate of 3–5 ml/s) (27). Multi-planar reconstruction (MPR) images help to identify new types of DIVC, and 3D images provide an accurate diagnosis. MR is also an important diagnostic aid and is suitable for children and pregnant women because of its lack of radiation exposure. DIVC is easily misdiagnosed as enlarged lymph nodes, abnormally dilated veins, dilated left renal pelvis ureter, atypical retroperitoneal cyst, or small bowel collaterals (28). When contrast enhancement is inadequate, DIVC is easily confused with enlarged retroperitoneal lymph nodes and misdiagnosed as lymph node metastases from a malignancy (29). We suspected RDIVC with dilated effusion or abnormal course of the right ureter and formation of the interiliac veins may be used as differential diagnoses. The imaging report of the patient failed definitively diagnose RDIVC, and the radiologist should have a better understanding of DIVC to guide clinical diagnosis and treatment.

## Conclusion

The formation of both ventrally and dorsally aligned DIVC is rarer. The infrarenal segment with DIVC is a relatively rare congenital abnormality that presents some diagnostic difficulties in imaging. It should be clarified the effects of DIVC on DVT formation, and the importance of imaging for preoperative planning. Awareness of the composition of IVC and the embryonic developmental mechanisms of the DIVC facilitates the diagnosis and the interventional treatment of associated venous disease, avoiding misdiagnosis and reducing the incidence of complications.

## Data Availability

The raw data supporting the conclusions of this article will be made available by the authors, without undue reservation.
